# (*E*)-*N*′-{7-Meth­oxy­spiro­[chromeno[4,3-*d*]thia­zole-4,1′-cyclo­hexa­n]-2-yl}-*N*,*N*-dimethyl­acetimidamide

**DOI:** 10.1107/S1600536811040359

**Published:** 2011-10-05

**Authors:** Kamini Kapoor, Vivek K. Gupta, Rajni Kant, Poorvesh M. Vyas, Mihir J. Joshi, Kalpesh M. Menpara, Kartik D. Ladva

**Affiliations:** a X-ray Crystallography Laboratory, Post-Graduate Department of Physics, University of Jammu, Jammu Tawi 180 006, India; b Physics Department, Saurashtra University, Rajkot 360 005, India; c Shri M. N. Virani Science College, Rajkot 360 005, India

## Abstract

In the chromenothia­zole ring system of the title mol­ecule, C_20_H_25_N_3_O_2_S, the pyran ring is in a half-chair conformation. The dihedral angle between the thia­zole and benzene rings is 14.78 (6)°. The cyclo­hexane ring is in a chair conformation. The crystal structure is stabilized by weak inter­molecular C—H⋯N and C—H⋯O hydrogen bonds.

## Related literature

For the biological activity of heterocyclic compounds containing nitro­gen and sulfur, see: Bishayee *et al.* (1997 ▶); Cruz *et al.* (1995 ▶); Chitamber & Wereley (1997 ▶). For the biological activity of thiazoles, see: Pawar *et al.* (2009 ▶). Schiff bases and acetamidine play an important role in many biological processes and are of great importance for the preparation of various pharmaceuticals, see: More *et al.* (2001 ▶); Sutariya *et al.* (2007 ▶); Murza *et al.* (1999 ▶); Dong *et al.* (2006 ▶); Jayashree *et al.* (2005 ▶); Modi *et al.* (1971 ▶); Vicini *et al.* (2003 ▶). For standard bond-length data, see: Allen *et al.* (1987 ▶). For ring conformations, see: Duax & Norton (1975 ▶).
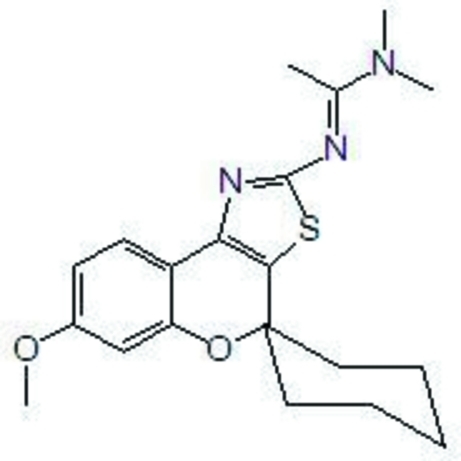


## Experimental

### 

#### Crystal data

C_20_H_25_N_3_O_2_S
*M* *_r_* = 371.49
Monoclinic, 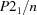

*a* = 9.2510 (2) Å
*b* = 20.0273 (4) Å
*c* = 10.7301 (2) Å
β = 90.840 (2)°
*V* = 1987.78 (7) Å^3^
*Z* = 4
Mo *K*α radiation
μ = 0.18 mm^−1^
*T* = 293 K
0.3 × 0.2 × 0.1 mm

#### Data collection

Oxford Diffraction Xcalibur Sapphire3 diffractometer
Absorption correction: multi-scan (*CrysAlis RED*; Oxford Diffraction, 2009 ▶) *T*_min_ = 0.892, *T*_max_ = 1.000
56290 measured reflections
3482 independent reflections
2835 reflections with *I* > 2σ(*I*)
*R*_int_ = 0.041

#### Refinement

*R*[*F* ^2^ > 2σ(*F* ^2^)] = 0.043
*wR*(*F* ^2^) = 0.113
*S* = 1.02
3482 reflections
291 parameters
H atoms treated by a mixture of independent and constrained refinement
Δρ_max_ = 0.18 e Å^−3^
Δρ_min_ = −0.19 e Å^−3^

### 

Data collection: *CrysAlis PRO* (Oxford Diffraction, 2009 ▶); cell refinement: *CrysAlis PRO*; data reduction: *CrysAlis PRO*; program(s) used to solve structure: *SHELXS97* (Sheldrick, 2008 ▶); program(s) used to refine structure: *SHELXL97* (Sheldrick, 2008 ▶); molecular graphics: *ORTEP-3* (Farrugia, 1997 ▶); software used to prepare material for publication: *PLATON* (Spek, 2009 ▶) and *PARST* (Nardelli, 1995 ▶).

## Supplementary Material

Crystal structure: contains datablock(s) I, global. DOI: 10.1107/S1600536811040359/lh5340sup1.cif

Structure factors: contains datablock(s) I. DOI: 10.1107/S1600536811040359/lh5340Isup2.hkl

Additional supplementary materials:  crystallographic information; 3D view; checkCIF report

## Figures and Tables

**Table 1:** Hydrogen-bond geometry (Å, °)

*D*—H⋯*A*	*D*—H	H⋯*A*	*D*⋯*A*	*D*—H⋯*A*
C19—H19*B*⋯O5^i^	0.96	2.41	3.335 (3)	161
C6—H61⋯N1^ii^	0.95 (2)	2.59 (2)	3.441 (3)	149.3 (15)

Symmetry codes: (i) 
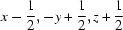
; (ii) 
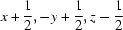
.

**Fig. 1.**
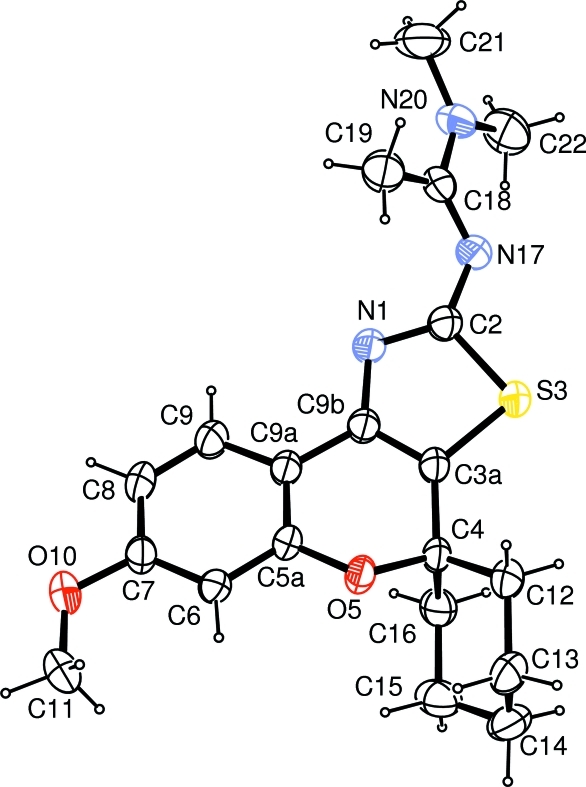
The molecular sreucture with ellipsoids are drawn at the 40% probability level. H atoms are shown as small spheres of arbitrary radii.

**Fig. 2.**
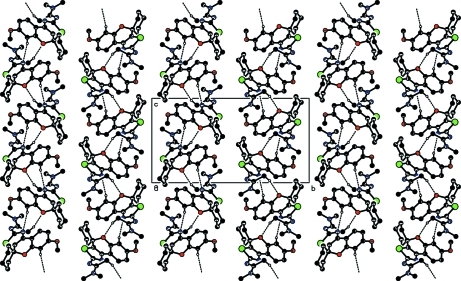
The packing arrangement of molecules. The broken lines show weak intermolecular hydrogen bonds.

## supplementary crystallographic information

### Comment

Heterocyclic compounds containing nitrogen and sulfur are used for medical purposes for the treatment of different kinds of fungal and bacterial infection along with treatment of e.g. gastric ulcers and cancer (Bishayee *et al.*, 1997; Cruz *et al.*, 1995; Chitamber & Wereley, 1997). Thiazoles exhibit a wide range of biological activities (Pawar *et al.*, 2009). Schiff bases and acetamidine play an important role in many biological processes. They are of great importance for the preparation of various pharmaceuticals and are used in many other areas of chemistry as starting materials. Their facile synthesis and numerous biological activities contribute greatly to their Schiff bases and acetamidine popularity (More *et al.*, 2001; Sutariya *et al.*, 2007; Murza *et al.*, 1999; Dong *et al.*, 2006; Jayashree *et al.*, 2005; Modi *et al.*, 1971; Vicini *et al.*, 2003). Therefore, Schiff bases and acetamidine of amino thiazoles are expected to be biologically active. We report herein the X-ray crystallographic studies of a novel acetamidine base derived from substituted 8-methoxyspiro[chromeno[4,3-*d*] [1,3]thiazole-4,1-cyclohexan]-2-amine as a possible hybrid antimicrobial agent. In the title compound (Fig. 1), the methoxy substituent at the C7 atom forms the torsion angle of 178.4 (2) ° [(+) antiperiplanar conformation] with the atom set O10/C7/C8/C9. The benzopyran ring has a half-chair conformation with asymmetry parameter: ΔC2(C4—O5) = 4.49 (Duax *et al.*, 1975). The cyclohexane ring has a chair conformation. The asymmetry parameters are: ΔCs(C4)=0.24; ΔC2(C4—C12)= 0.88. The dihedral angle between the best least squares planes through the thiazole and  benzene rings is 14.75 (7)°. The stabilization of crystal packing (Fig. 2) is influenced by intermolecular C—H···N and C—H···O hydrogen bonding [C6—H61···N1 (symmetry code: *x* + 1/2, -*y* + 1/2, *z* - 1/2); C19—H19B···O5 (symmetry code: *x* - 1/2, -*y* + 1/2, *z* + 1/2)]. These interactions link the molecules into chains that run parallel to [-1 0 1].

### Experimental

An ice cold solution of phosphorus oxychloride (0.85 mmol) in dry toluene(20 ml) was added to the suitable amount of acetamide (0.47 mmol), and the mixture was stirred for 30 min at room temperature. At the end of the reaction, 7-methoxyspiro[4,3-*d*][1,3]thiazole-4,1-cyclohexan]-2-amine (0.42 mmol) dissolved in dry toluene was added drop wise and the reaction mixture was refluxed for 6 h. The solution was then cooled, carefully poured into the ice-water, and made alkaline with 1 N NaOH solution. The organic layer was extracted with CHCl_3_, washed to neutrality with water, dried over sodium sulfate, filtered and then evaporated *in vacuo*. The crude material was purified by column chromatography on silica gel eluting with a hexane/ethyl acetate(7:3) mixture. Single crystals suitable for X-ray measurements were obtained by crystallization from CHCl_3_ at room temperature.

### Refinement

All H atoms (except methyl H atoms) were located in a difference Fourier map and refined freely. Methyl H atoms were positioned geometrically and refined using a riding model with C—H = 0.96 Å. The *U*_iso_(H) values were constrained to be 1.5U_eq_(C methyl).

### Crystal data

**Table:**

C_20_H_25_N_3_O_2_S	*F*(000) = 792
*M**_r_* = 371.49	*D*_x_ = 1.241 Mg m^−^^3^
Monoclinic, *P*2_1_/*n*	Mo *K*α radiation, λ = 0.71073 Å
Hall symbol: -P 2yn	Cell parameters from 24440 reflections
*a* = 9.2510 (2) Å	θ = 3.5–29.0°
*b* = 20.0273 (4) Å	µ = 0.18 mm^−^^1^
*c* = 10.7301 (2) Å	*T* = 293 K
β = 90.840 (2)°	Plate, light-brown
*V* = 1987.78 (7) Å^3^	0.3 × 0.2 × 0.1 mm
*Z* = 4	

### Data collection

**Table:**

Oxford Diffraction Xcalibur Sapphire3 diffractometer	3482 independent reflections
Radiation source: fine-focus sealed tube	2835 reflections with *I* > 2σ(*I*)
graphite	*R*_int_ = 0.041
Detector resolution: 16.1049 pixels mm^-1^	θ_max_ = 25.0°, θ_min_ = 3.5°
ω scans	*h* = −11→11
Absorption correction: multi-scan (*CrysAlis RED*; Oxford Diffraction, 2009)	*k* = −23→23
*T*_min_ = 0.892, *T*_max_ = 1.000	*l* = −12→12
56290 measured reflections	

### Refinement

**Table:**

Refinement on *F*^2^	Primary atom site location: structure-invariant direct methods
Least-squares matrix: full	Secondary atom site location: difference Fourier map
*R*[*F*^2^ > 2σ(*F*^2^)] = 0.043	Hydrogen site location: inferred from neighbouring sites
*wR*(*F*^2^) = 0.113	H atoms treated by a mixture of independent and constrained refinement
*S* = 1.02	*w* = 1/[σ^2^(*F*_o_^2^) + (0.0521*P*)^2^ + 0.884*P*] where *P* = (*F*_o_^2^ + 2*F*_c_^2^)/3
3482 reflections	(Δ/σ)_max_ = 0.001
291 parameters	Δρ_max_ = 0.18 e Å^−^^3^
0 restraints	Δρ_min_ = −0.19 e Å^−^^3^

### Special details

**Table:**

Experimental. *CrysAlis PRO*, Oxford Diffraction Ltd., Version 1.171.34.40 (release 27–08-2010 CrysAlis171. NET) (compiled Aug 27 2010,11:50:40) Empirical absorption correction using spherical harmonics, implemented in SCALE3 ABSPACK scaling algorithm.
Geometry. All e.s.d.'s (except the e.s.d. in the dihedral angle between two l.s. planes) are estimated using the full covariance matrix. The cell e.s.d.'s are taken into account individually in the estimation of e.s.d.'s in distances, angles and torsion angles; correlations between e.s.d.'s in cell parameters are only used when they are defined by crystal symmetry. An approximate (isotropic) treatment of cell e.s.d.'s is used for estimating e.s.d.'s involving l.s. planes.
Refinement. Refinement of *F*^2^ against ALL reflections. The weighted *R*-factor *wR* and goodness of fit *S* are based on *F*^2^, conventional *R*-factors *R* are based on *F*, with *F* set to zero for negative *F*^2^. The threshold expression of *F*^2^ > σ(*F*^2^) is used only for calculating *R*-factors(gt) *etc*. and is not relevant to the choice of reflections for refinement. *R*-factors based on *F*^2^ are statistically about twice as large as those based on *F*, and *R*- factors based on ALL data will be even larger.

### Fractional atomic coordinates and isotropic or equivalent isotropic displacement parameters (Å^2^)

**Table:**

	*x*	*y*	*z*	*U*_iso_*/*U*_eq_	
N1	0.33437 (18)	0.18039 (8)	0.41748 (15)	0.0474 (4)	
C2	0.2587 (2)	0.12626 (10)	0.39594 (19)	0.0467 (5)	
C3A	0.4732 (2)	0.12918 (9)	0.26463 (18)	0.0428 (5)	
S3	0.33236 (6)	0.07336 (3)	0.28200 (5)	0.05248 (19)	
C4	0.6032 (2)	0.12315 (9)	0.18332 (17)	0.0410 (4)	
O5	0.64694 (14)	0.19099 (6)	0.14928 (11)	0.0428 (3)	
C5A	0.65071 (19)	0.23902 (8)	0.24074 (16)	0.0368 (4)	
C6	0.7445 (2)	0.29214 (9)	0.22330 (18)	0.0395 (4)	
C7	0.7481 (2)	0.34279 (9)	0.3113 (2)	0.0459 (5)	
C8	0.6590 (3)	0.34051 (11)	0.4146 (2)	0.0551 (6)	
C9	0.5632 (3)	0.28851 (11)	0.4276 (2)	0.0539 (5)	
C9A	0.5568 (2)	0.23671 (9)	0.34111 (17)	0.0419 (4)	
C9B	0.4543 (2)	0.18148 (9)	0.34258 (17)	0.0421 (4)	
O10	0.83754 (17)	0.39732 (7)	0.30446 (15)	0.0619 (4)	
C11	0.9325 (3)	0.40072 (13)	0.2026 (3)	0.0766 (8)	
H11A	0.9927	0.3617	0.2020	0.115*	
H11B	0.9919	0.4398	0.2104	0.115*	
H11C	0.8772	0.4029	0.1262	0.115*	
C12	0.5715 (3)	0.08891 (13)	0.0596 (2)	0.0562 (6)	
C13	0.7053 (3)	0.08381 (14)	−0.0209 (3)	0.0693 (7)	
C14	0.8293 (3)	0.05035 (14)	0.0475 (3)	0.0818 (9)	
C15	0.8642 (3)	0.08530 (14)	0.1699 (3)	0.0693 (7)	
C16	0.7304 (2)	0.08994 (12)	0.2511 (2)	0.0535 (5)	
N17	0.13926 (19)	0.10532 (9)	0.45830 (17)	0.0537 (5)	
C18	0.0370 (2)	0.14748 (11)	0.4842 (2)	0.0501 (5)	
C19	0.0168 (3)	0.21331 (12)	0.4193 (2)	0.0657 (6)	
H19A	0.0794	0.2155	0.3489	0.099*	
H19B	0.0400	0.2489	0.4761	0.099*	
H19C	−0.0818	0.2176	0.3915	0.099*	
N20	−0.06057 (19)	0.12955 (11)	0.56983 (19)	0.0650 (5)	
C21	−0.1802 (3)	0.17228 (18)	0.6067 (3)	0.0986 (11)	
H21A	−0.1614	0.2174	0.5812	0.148*	
H21B	−0.1900	0.1707	0.6956	0.148*	
H21C	−0.2680	0.1569	0.5675	0.148*	
C22	−0.0438 (4)	0.06674 (15)	0.6362 (3)	0.0923 (10)	
H22A	−0.1023	0.0331	0.5963	0.139*	
H22B	−0.0739	0.0723	0.7208	0.139*	
H22C	0.0558	0.0533	0.6352	0.139*	
H61	0.803 (2)	0.2918 (9)	0.1512 (18)	0.044 (5)*	
H81	0.664 (2)	0.3751 (11)	0.477 (2)	0.058 (6)*	
H91	0.499 (3)	0.2877 (11)	0.494 (2)	0.064 (7)*	
H161	0.698 (2)	0.0457 (12)	0.275 (2)	0.061 (6)*	
H162	0.752 (2)	0.1132 (11)	0.328 (2)	0.058 (6)*	
H121	0.537 (2)	0.0441 (12)	0.079 (2)	0.061 (6)*	
H122	0.493 (3)	0.1108 (13)	0.017 (2)	0.076 (8)*	
H131	0.679 (3)	0.0603 (13)	−0.093 (3)	0.076 (8)*	
H132	0.734 (3)	0.1291 (13)	−0.046 (2)	0.063 (7)*	
H141	0.913 (3)	0.0497 (14)	−0.006 (3)	0.092 (9)*	
H142	0.801 (3)	0.0054 (16)	0.064 (3)	0.093 (9)*	
H151	0.942 (3)	0.0618 (14)	0.216 (3)	0.092 (9)*	
H152	0.897 (3)	0.1321 (13)	0.153 (2)	0.068 (7)*	

### Atomic displacement parameters (Å^2^)

**Table:**

	*U*^11^	*U*^22^	*U*^33^	*U*^12^	*U*^13^	*U*^23^
N1	0.0512 (10)	0.0403 (9)	0.0512 (10)	−0.0060 (8)	0.0149 (8)	−0.0058 (7)
C2	0.0484 (11)	0.0394 (11)	0.0525 (12)	−0.0010 (9)	0.0098 (9)	−0.0008 (9)
C3A	0.0480 (11)	0.0324 (10)	0.0482 (11)	−0.0054 (8)	0.0072 (9)	−0.0044 (8)
S3	0.0512 (3)	0.0383 (3)	0.0684 (4)	−0.0089 (2)	0.0148 (3)	−0.0121 (2)
C4	0.0488 (11)	0.0285 (9)	0.0460 (11)	−0.0059 (8)	0.0086 (8)	−0.0056 (8)
O5	0.0580 (8)	0.0311 (7)	0.0395 (7)	−0.0040 (6)	0.0085 (6)	−0.0047 (5)
C5A	0.0429 (10)	0.0281 (9)	0.0393 (10)	0.0018 (7)	0.0003 (8)	−0.0033 (7)
C6	0.0408 (10)	0.0331 (10)	0.0446 (11)	0.0024 (8)	0.0035 (8)	0.0001 (8)
C7	0.0455 (11)	0.0329 (10)	0.0592 (12)	−0.0027 (8)	−0.0015 (9)	−0.0045 (9)
C8	0.0649 (14)	0.0389 (11)	0.0617 (13)	−0.0051 (10)	0.0081 (11)	−0.0193 (10)
C9	0.0628 (14)	0.0459 (12)	0.0534 (12)	−0.0069 (10)	0.0144 (11)	−0.0144 (10)
C9A	0.0479 (11)	0.0340 (10)	0.0438 (10)	−0.0021 (8)	0.0053 (8)	−0.0050 (8)
C9B	0.0475 (11)	0.0362 (10)	0.0428 (10)	−0.0028 (8)	0.0075 (8)	−0.0027 (8)
O10	0.0637 (10)	0.0431 (8)	0.0791 (11)	−0.0182 (7)	0.0083 (8)	−0.0145 (7)
C11	0.0684 (16)	0.0602 (15)	0.102 (2)	−0.0289 (13)	0.0216 (15)	−0.0149 (14)
C12	0.0602 (14)	0.0509 (14)	0.0579 (13)	−0.0119 (11)	0.0102 (11)	−0.0201 (11)
C13	0.0826 (18)	0.0614 (16)	0.0645 (16)	−0.0119 (14)	0.0242 (14)	−0.0268 (13)
C14	0.0795 (19)	0.0494 (15)	0.118 (3)	0.0057 (14)	0.0509 (19)	−0.0093 (15)
C15	0.0494 (14)	0.0596 (16)	0.099 (2)	0.0071 (12)	0.0135 (13)	0.0161 (15)
C16	0.0531 (13)	0.0411 (12)	0.0665 (15)	−0.0008 (10)	0.0051 (11)	0.0067 (11)
N17	0.0493 (10)	0.0446 (10)	0.0677 (11)	−0.0064 (8)	0.0177 (9)	−0.0025 (9)
C18	0.0402 (11)	0.0526 (12)	0.0575 (12)	−0.0095 (9)	0.0004 (9)	−0.0127 (10)
C19	0.0538 (13)	0.0627 (15)	0.0801 (16)	0.0048 (11)	−0.0125 (12)	−0.0052 (13)
N20	0.0440 (10)	0.0762 (14)	0.0753 (13)	−0.0106 (9)	0.0150 (9)	−0.0155 (11)
C21	0.0528 (16)	0.137 (3)	0.106 (2)	0.0069 (17)	0.0207 (15)	−0.033 (2)
C22	0.092 (2)	0.086 (2)	0.100 (2)	−0.0273 (17)	0.0405 (18)	0.0049 (17)

### Geometric parameters (Å, °)

**Table:**

N1—C2	1.309 (3)	C12—H121	0.97 (2)
N1—C9B	1.380 (2)	C12—H122	0.96 (3)
C2—N17	1.366 (2)	C13—C14	1.510 (4)
C2—S3	1.762 (2)	C13—H131	0.94 (3)
C3A—C9B	1.353 (3)	C13—H132	0.98 (2)
C3A—C4	1.501 (3)	C14—C15	1.519 (4)
C3A—S3	1.7292 (19)	C14—H141	0.97 (3)
C4—O5	1.465 (2)	C14—H142	0.95 (3)
C4—C12	1.519 (3)	C15—C16	1.527 (3)
C4—C16	1.526 (3)	C15—H151	0.98 (3)
O5—C5A	1.374 (2)	C15—H152	1.00 (3)
C5A—C6	1.387 (3)	C16—H161	0.97 (2)
C5A—C9A	1.394 (3)	C16—H162	0.97 (2)
C6—C7	1.386 (3)	N17—C18	1.301 (3)
C6—H61	0.95 (2)	C18—N20	1.346 (3)
C7—O10	1.373 (2)	C18—C19	1.501 (3)
C7—C8	1.391 (3)	C19—H19A	0.9600
C8—C9	1.376 (3)	C19—H19B	0.9600
C8—H81	0.96 (2)	C19—H19C	0.9600
C9—C9A	1.393 (3)	N20—C22	1.452 (4)
C9—H91	0.94 (2)	N20—C21	1.458 (3)
C9A—C9B	1.457 (3)	C21—H21A	0.9600
O10—C11	1.414 (3)	C21—H21B	0.9600
C11—H11A	0.9600	C21—H21C	0.9600
C11—H11B	0.9600	C22—H22A	0.9600
C11—H11C	0.9600	C22—H22B	0.9600
C12—C13	1.523 (3)	C22—H22C	0.9600
			
C2—N1—C9B	110.04 (16)	C14—C13—C12	111.8 (2)
N1—C2—N17	127.00 (18)	C14—C13—H131	111.6 (16)
N1—C2—S3	114.16 (14)	C12—C13—H131	107.3 (16)
N17—C2—S3	118.62 (15)	C14—C13—H132	109.5 (14)
C9B—C3A—C4	122.24 (17)	C12—C13—H132	108.6 (14)
C9B—C3A—S3	109.23 (14)	H131—C13—H132	108 (2)
C4—C3A—S3	128.48 (14)	C13—C14—C15	111.5 (2)
C3A—S3—C2	89.18 (9)	C13—C14—H141	109.2 (17)
O5—C4—C3A	107.26 (14)	C15—C14—H141	110.7 (16)
O5—C4—C12	104.56 (16)	C13—C14—H142	107.4 (17)
C3A—C4—C12	113.46 (17)	C15—C14—H142	109.4 (18)
O5—C4—C16	108.01 (16)	H141—C14—H142	109 (2)
C3A—C4—C16	112.13 (17)	C14—C15—C16	110.9 (2)
C12—C4—C16	110.92 (18)	C14—C15—H151	110.9 (17)
C5A—O5—C4	118.37 (13)	C16—C15—H151	109.5 (17)
O5—C5A—C6	116.73 (16)	C14—C15—H152	109.6 (14)
O5—C5A—C9A	121.25 (16)	C16—C15—H152	107.3 (14)
C6—C5A—C9A	121.85 (16)	H151—C15—H152	109 (2)
C7—C6—C5A	118.55 (18)	C4—C16—C15	112.4 (2)
C7—C6—H61	123.5 (12)	C4—C16—H161	106.8 (13)
C5A—C6—H61	117.9 (12)	C15—C16—H161	110.5 (13)
O10—C7—C6	123.63 (18)	C4—C16—H162	110.4 (13)
O10—C7—C8	115.69 (17)	C15—C16—H162	110.8 (13)
C6—C7—C8	120.68 (18)	H161—C16—H162	105.7 (19)
C9—C8—C7	119.71 (19)	C18—N17—C2	120.09 (18)
C9—C8—H81	120.0 (13)	N17—C18—N20	118.0 (2)
C7—C8—H81	120.3 (13)	N17—C18—C19	123.8 (2)
C8—C9—C9A	121.1 (2)	N20—C18—C19	118.1 (2)
C8—C9—H91	120.6 (14)	C18—C19—H19A	109.5
C9A—C9—H91	118.2 (15)	C18—C19—H19B	109.5
C9—C9A—C5A	118.01 (18)	H19A—C19—H19B	109.5
C9—C9A—C9B	125.40 (18)	C18—C19—H19C	109.5
C5A—C9A—C9B	116.51 (16)	H19A—C19—H19C	109.5
C3A—C9B—N1	117.40 (17)	H19B—C19—H19C	109.5
C3A—C9B—C9A	119.39 (17)	C18—N20—C22	119.9 (2)
N1—C9B—C9A	123.18 (16)	C18—N20—C21	123.2 (2)
C7—O10—C11	117.49 (17)	C22—N20—C21	116.8 (2)
O10—C11—H11A	109.5	N20—C21—H21A	109.5
O10—C11—H11B	109.5	N20—C21—H21B	109.5
H11A—C11—H11B	109.5	H21A—C21—H21B	109.5
O10—C11—H11C	109.5	N20—C21—H21C	109.5
H11A—C11—H11C	109.5	H21A—C21—H21C	109.5
H11B—C11—H11C	109.5	H21B—C21—H21C	109.5
C4—C12—C13	112.2 (2)	N20—C22—H22A	109.5
C4—C12—H121	106.7 (13)	N20—C22—H22B	109.5
C13—C12—H121	109.2 (13)	H22A—C22—H22B	109.5
C4—C12—H122	110.2 (16)	N20—C22—H22C	109.5
C13—C12—H122	112.2 (16)	H22A—C22—H22C	109.5
H121—C12—H122	106 (2)	H22B—C22—H22C	109.5
			
C9B—N1—C2—N17	174.7 (2)	C4—C3A—C9B—N1	−176.95 (18)
C9B—N1—C2—S3	0.3 (2)	S3—C3A—C9B—N1	0.5 (2)
C9B—C3A—S3—C2	−0.26 (16)	C4—C3A—C9B—C9A	5.1 (3)
C4—C3A—S3—C2	176.97 (19)	S3—C3A—C9B—C9A	−177.50 (15)
N1—C2—S3—C3A	0.00 (17)	C2—N1—C9B—C3A	−0.5 (3)
N17—C2—S3—C3A	−174.98 (18)	C2—N1—C9B—C9A	177.41 (18)
C9B—C3A—C4—O5	−32.2 (3)	C9—C9A—C9B—C3A	−170.1 (2)
S3—C3A—C4—O5	150.85 (15)	C5A—C9A—C9B—C3A	13.4 (3)
C9B—C3A—C4—C12	−147.2 (2)	C9—C9A—C9B—N1	12.1 (3)
S3—C3A—C4—C12	35.9 (3)	C5A—C9A—C9B—N1	−164.47 (18)
C9B—C3A—C4—C16	86.2 (2)	C6—C7—O10—C11	−1.2 (3)
S3—C3A—C4—C16	−90.7 (2)	C8—C7—O10—C11	178.4 (2)
C3A—C4—O5—C5A	44.4 (2)	O5—C4—C12—C13	63.2 (3)
C12—C4—O5—C5A	165.17 (16)	C3A—C4—C12—C13	179.8 (2)
C16—C4—O5—C5A	−76.6 (2)	C16—C4—C12—C13	−53.0 (3)
C4—O5—C5A—C6	154.00 (16)	C4—C12—C13—C14	54.4 (3)
C4—O5—C5A—C9A	−30.6 (2)	C12—C13—C14—C15	−55.2 (3)
O5—C5A—C6—C7	177.88 (16)	C13—C14—C15—C16	55.1 (3)
C9A—C5A—C6—C7	2.6 (3)	O5—C4—C16—C15	−60.5 (2)
C5A—C6—C7—O10	179.29 (18)	C3A—C4—C16—C15	−178.52 (19)
C5A—C6—C7—C8	−0.3 (3)	C12—C4—C16—C15	53.5 (3)
O10—C7—C8—C9	178.4 (2)	C14—C15—C16—C4	−54.7 (3)
C6—C7—C8—C9	−2.0 (3)	N1—C2—N17—C18	45.0 (3)
C7—C8—C9—C9A	2.1 (4)	S3—C2—N17—C18	−140.69 (18)
C8—C9—C9A—C5A	0.1 (3)	C2—N17—C18—N20	−164.45 (19)
C8—C9—C9A—C9B	−176.4 (2)	C2—N17—C18—C19	18.9 (3)
O5—C5A—C9A—C9	−177.56 (18)	N17—C18—N20—C22	4.1 (3)
C6—C5A—C9A—C9	−2.4 (3)	C19—C18—N20—C22	−179.0 (2)
O5—C5A—C9A—C9B	−0.7 (3)	N17—C18—N20—C21	−179.9 (2)
C6—C5A—C9A—C9B	174.37 (17)	C19—C18—N20—C21	−3.1 (3)

### Hydrogen-bond geometry (Å, °)

**Table:**

*D*—H···*A*	*D*—H	H···*A*	*D*···*A*	*D*—H···*A*
C19—H19B···O5^i^	0.96	2.41	3.335 (3)	161
C6—H61···N1^ii^	0.95 (2)	2.59 (2)	3.441 (3)	149.3 (15)

Symmetry codes: (i) *x*−1/2, −*y*+1/2, *z*+1/2; (ii) *x*+1/2, −*y*+1/2, *z*−1/2.
